# Supplementary material for: Are carbon emissions trading and green financial instruments synergistic? -Comprehensive quantitative research based on content analysis
Source: PLoS One. 2024 Mar 7;19(3):e0298601. doi: 10.1371/journal.pone.0298601 (PMC10919601; doi:10.1371/journal.pone.0298601)
Supplement: S1 Data — (ZIP) [file pone.0298601.s001.zip › Data/地级市Z_F工作报告.zip!/╣π╢1⁄2/╓Θ║ú╩╨/└┤╫╘╣1⁄2╓┌║┼í╛┬φ┐╦╩2╛▌═°í┐_2011.docx]

各位代表：

　　现在，我代表市人民政府向大会作政府工作报告，请予审议，并请各位政协委员和其他列席人员提出意见。

　　一、2010年主要工作

　　刚刚过去的2010年，恰逢经济特区成立30周年，是我市抓机遇、谋发展、促升温取得显著成绩的一年。在党中央、国务院和省委、省政府以及市委的正确领导下，在市人大及其常委会和市政协的监督支持下，市政府坚持以邓小平理论和“三个代表”重要思想为指导，深入贯彻落实科学发展观，全面实施《珠江三角洲地区改革发展规划纲要（2008—2020年）》（以下简称珠三角《规划纲要》）和《横琴总体发展规划》，大力推进“三促进一保持”（促进提高自主创新能力、促进传统产业转型升级、促进建设现代产业体系、保持经济平稳较快增长），一心一意谋发展，脚踏实地惠民生，推动我市经济社会迈出科学发展新步伐，为建设幸福珠海奠定了新的坚实基础。

　　（一）积极应对形势变化，经济保持平稳较快增长。

　　综合经济实力明显增强。面对复杂多变的内外部环境，我们积极应对，科学决策，早预见、快行动，及时出台了扩大内需、稳定外贸、调整结构、加大投资等一揽子政策措施，促进全市经济平稳较快发展。全市生产总值达1202.6亿元，比上年增长12.8%，增速提高了6.2个百分点；来源于全市的财税总收入（不含土地出让收入）达393.1亿元，增长28.7%；地方财政一般预算收入124.5亿元，增长22.8%；年末全市金融机构存贷款余额分别达2748.7亿元和1472.5亿元，各增长30.6%和38.6%，增速居全省首位。

　　重大项目建设大力推进。强化项目推进机制，加快实施“十大重点建设工程”，一批大项目相继动工或建成投产。市政府投资完成82.1亿元，完成年度计划的102.4%，比上年增长13.6%。全社会固定资产投资完成501.5亿元，增长22.2%。港珠澳大桥人工岛填海工程、广珠铁路、高栏港高速、机场高速、拱北口岸改建等工程加快推进。广珠城轨广州南站至珠海北站开通，珠海大道辅道、港湾大道等6条道路改造完成。珠海大道主道改造和金港高速金凤段等项目动工。香海路、广珠城轨延长线等项目前期工作加快开展。高栏港7万吨级主航道扩建工程完工，10万吨级主航道和集装箱、矿石、煤炭等大型码头泊位加快建设。一批总投资达140多亿元的重大工业项目动工建设。一批工业项目建成投产，预计总产值达100多亿元。电网工程完成投资13.7亿元，风力、天然气发电等项目加快推进，电力保障能力明显增强。

　　扩内需稳外贸成效突出。深入实施扩大内需战略，组织企业参加内销展会16场次，成交金额超过254亿元。投入2758万元鼓励加工贸易企业扩大产品内销，全年内销总额达882.8亿元，比上年增长38.7%。累计销售家电下乡、以旧换新产品12.3万台，销售额达4.4亿元，新增国家和省级万村千乡农家店11个。社会消费品零售总额486亿元，增长20.5%。着力促进外经贸企业平稳发展，全年外贸进出口总额达434.8亿美元，增长16.1%，其中出口208.6亿美元，增长17.3%。

　　（二）加快调整产业结构，现代产业体系建设扎实推进。

　　自主创新能力提高。推动出台《珠海经济特区科技创新促进条例》，深入贯彻自主创新行动计划，全面提升自主创新能力。全市财政一般预算安排科技支出5.7亿元，比上年增长44.4%。高新技术产品产值达1250亿元，增长18.5%，占工业总产值的比重达41%。深化产学研合作，30个产学研合作项目获得省财政专项资助，金额比上年增加一倍以上。新增省部产学研结合示范基地、科技创新平台、特派员工作站12个和科技特派员85名。鼓励企业创新，新增国家高新技术企业43家、省百强创新型企业8家、省级自主创新产品76件。全年专利申请量、授权量分别增长27.9%和37.9%。“以质取胜”、“名牌带动”和“技术标准”工作深入开展，新增省级以上名牌名标27个，总数达158个。

　　产业结构升级加快。坚持现代服务业和先进制造业“双轮驱动”，大力推进现代产业体系建设，产业规模和企业效益进一步提升。全市规模以上工业总产值和增加值分别达2985.9亿元和615.4亿元，比上年增长16.3%和18.2%。全市规模以上工业企业实现利润163.6亿元，增长45.1%，经济效益指数达174.6%，比上年提升18.8个百分点。先进制造业发展壮大，第二产业对全市生产总值增长的贡献率达74.8%，电子信息、生物医药等六大优势产业增加值占全市工业增加值的77.2%，23个制造业项目入选省现代产业500强。全市制造业完成投资105.6亿元，增长28.2%，扭转了近年来制造业投资持续下滑的状况。现代服务业加快发展，实现增加值282.9亿元，占全市服务业增加值比重达55.4%。成功举办第八届中国国际航空航天博览会，签约项目28个，成交金额93亿美元，为历届航展之最。港口货物吞吐量、机场旅客吞吐量分别为6056万吨和181.9万人次，各增长37.4%和31.3%。软件业加速发展，软件产业总收入达185亿元，增长25%。旅游业总收入达219.4亿元，增长29.9%。出台战略性新兴产业发展专项政策，全市各级财政投入约31.8亿元扶持战略性新兴产业项目，帮助战略性新兴产业企业争取到国家和省扶持资金4.1亿元。软件和集成电路设计产业基地入选省首批战略性新兴产业基地，5家企业成为国家规划布局内的重点软件企业，12家企业入选省战略性新兴产业骨干企业和培育企业。民营经济加快发展，全年实现增加值294.4亿元，增长10%。企业融资取得新进展，6家企业成功上市。通过“四位一体”（政府、银行、信用担保机构、企业）融资平台帮助221家企业融资12.2亿元，增长18.5%。荣获“转型?2010中国经济十大领军城市”称号。

　　招商引资成效明显。市区联动，形成合力，突出抓好重点区域和重点企业的招商引资工作。全年引进内资注册资本金80.1亿元，比上年增长41%，新注册内资项目484个，其中投资5000万元以上的项目24个。实际利用外资12.2亿美元，增长3.7%，其中制造业实际利用外资4.9亿美元，增长13.7%。新批外商直接投资项目212个，其中1000万美元以上的项目15个。目前已有39家世界500强企业、26家中央企业分别在我市投资85个项目和37个项目。荣获省“吸收外商直接投资综合一等奖”、“超额完成预期目标奖”等奖项。

　　节能减排扎实有效。强化节能减排工作责任制，加强节能减排监察执法，狠抓各项工作措施的落实。实施一批试点示范项目，大力发展循环经济，积极发展新能源和可再生能源。着力实施重点节能工程，投入1.8亿元支持重点节能项目，加快建设广东省绿色照明示范城市。推进污染减排设施建设，加强环境污染综合治理，依法关停70多家污染企业。全年单位生产总值能耗下降超过3.64%，化学需氧量、二氧化硫排放量分别控制在2.78万吨和3.3万吨以内，节能减排约束性指标全面完成。

　　（三）统筹推进城乡建设，城市化发展水平提高。

　　横琴新区建设强力推进。全力推进横琴新区开发建设，加大政策和财政支持力度，及时研究和协调解决横琴新区开发进程中的重大问题。市直12个部门向横琴新区授权97项，支持横琴新区行使市一级行政管理权限。横琴通关、财税、金融、产业等政策报批取得积极进展，基础设施和重大项目建设加快推进，实现了“一年有变化”的目标。

　　城区协调发展步伐加快。东部地区加快转型升级，全年共引进服务业投资75亿元，比上年增长117.9%；第三产业增加值达409.2亿元，占全市第三产业增加值的80.1%。大力推进“三旧”改造（旧城镇、旧厂房、旧村庄改造），启动“三旧”改造项目36个，涉及土地面积141.7万平方米，完成改造面积79.3万平方米，节地率达65%以上。西部中心城区开发建设启动，市财政对西部地区投入基建资金15.6亿元，比上年增长203.9%。西部地区生产总值、规模以上工业总产值占全市的比重分别为37.1%和51.5%，比上年各提高1个百分点。

　　农业农村经济稳定发展。统筹城乡发展，全面落实强农惠农政策。安排专项资金8000万元，扎实推进新农村建设。落实耕地保护责任，补充开发耕地785亩。投入2857.3万元全面开展农村水改工作，斗门区荔山村、南潮村和高新区东岸社区水改工程竣工。投资5670万元改造农村危桥6座，建设农村公路13条，总长25.9公里。推进强镇富民工作，井岸、南水、唐家湾、桂山、担杆、万山六个镇成为全省镇域经济综合发展力“百强镇”。现代生态农业加快发展，农业产业化水平不断提高，新增3个省级名牌农业类产品和2家省级农业龙头企业。“科技兴海”深入推进，海洋经济发展全面提速。

　　生态环境建设卓有成效。制定生态市建设规划和创建生态文明城市、宜居城乡行动纲要，全面落实生态建设“四个百分百”（工业项目百分百进园区、污水垃圾百分百达标处理、裸露山体百分百恢复绿化、节能减排百分百实现目标）行动方案。大力开展城市绿化美化活动，城市环境呈现新面貌。投入8443万元在城市道路和公园种植景观树，建成南屏橄榄园等4个特色公园。投入约3亿元建成82公里区域绿道和180公里城市社区绿道。扎实推进国家环保模范城市复查迎检工作，新建成10多项污水、垃圾处理设施，城市生活污水处理率达81%，比上年提高1个百分点；城镇生活垃圾无害化处理率达92.3%，提高6.2个百分点。在暨南大学珠海校区等地开展生活垃圾源头分类试点。开展全国生态文明试点城市建设，新增21所市级“绿色学校”、11个市级“绿色社区”、15个市级生态示范区。入选“中国十佳和谐可持续发展城市”、“中国十佳休闲城市”、世界特色魅力城市200强。

　　城市管理水平不断提高。加快城市总体规划修改工作，市区镇三级土地利用总体规划编制完成，城乡规划体系进一步完善。调整城市管理体制，推进城管执法重心下移，完成“数字城管”网络系统一期工程。坚持疏堵结合，利用迎亚运、办航展等有利契机，整治市容市貌，清拆各类违法户外广告设施6528平方米、违法建筑30.2万平方米。投入450万元支持3家农贸市场改造升级，改善农贸市场经营环境。投入1738万元改造横街小巷。加强道路交通安全综合治理，建设4座下沉式车行通道、20座地下人行通道、28个停车场802个停车位，连续七年被评为全国畅通工程一等管理水平城市。

　　（四）深化改革和区域合作，城市综合竞争力提升。

　　重点领域改革稳步开展。经济特区范围扩至全市，为我市推进改革先行先试、再创发展新优势开创了广阔空间。国有企业改革深入推进，董事会建设试点取得实效。13家市管企业营业收入达691.4亿元，比上年增长52.2%。完成第四轮行政审批制度改革，市级审批事项由648项减少到271项，减少了58%。赋予高栏港经济区、富山工业园依法行使部分市一级行政审批权限。推进社会管理体制改革先行先试，开展城市社区服务站、政府咨询和购买服务试点工作，城市基层社会治理和服务新体系初步建立。推行流动人口服务管理“一证通”制度（居住证制度），制定流动人口居住证积分入户政策。新医改稳步推进，集体林权制度改革等其他改革扎实开展。

　　区域交流合作深入开展。大力实施珠三角《规划纲要》，加强和完善区域合作机制，加快区域一体化进程。全面落实粤港、粤澳各项合作协议和优惠政策，推进珠港澳更紧密合作。全年对港澳进出口总额达71.8亿美元，比上年增长8.4%。实际利用港澳资金6.7亿美元，新增港澳企业161家，增长21.1%。竹银水源工程完成总投资的77%，竹洲头泵站及输水管线建成使用。深入实施珠三角五个一体化（环境保护、基础设施建设、城乡规划、产业布局和基本公共服务一体化）专项规划，珠中江三市新签人才服务、宣传文化等合作协议9项。试行珠中江公交“一卡通”结算方式，实行车辆通行费年票互认。

　　对口支援帮扶有效开展。“双转移”（“产业转移”和“劳动力转移”两大战略的统称。通过推动“双转移”工作，达到优化劳动力结构和人力资源配置，优化产业结构，促进区域协调发展的目的。）工作通过省考核，全年接收本省转移劳动力10350人。揭阳、茂名两个产业转移园新增项目15个，总投资12.5亿元；已建成投产项目33个，实现产值55.6亿元。开展“扶贫济困日”活动，募集社会捐款1亿元。全年投入揭阳市扶贫开发“双到”（规模到户、责任到人）资金1.4亿元，实施扶贫项目495个，实现脱贫8870户。落实市内34个经济欠发达村帮扶资金4940万元，帮扶项目144个。投入各类资金4.3亿元，圆满完成支援汶川绵虒镇27类工程99个项目建设任务。安排1100万元援建重庆市三县14个项目建设。

　　（五）大力改善民生，和谐社会建设步伐加快。

　　民生投入持续加大。全市财政用于民生方面的支出达57.6亿元，占一般预算支出的39.6%，比上年增长14.2%。启动实施惠民生办实事30项工程128个项目，涉及人口、住房、教育、卫生、就业、社会保障等领域，投资总额约148亿元。出台政策措施，加强价格调控监管，价格总水平保持基本稳定。人民生活水平进一步提高，城镇居民人均可支配收入25382元，增长11%；农村居民人均纯收入10187元，按可比口径增长10.8%。城乡居民储蓄存款余额982.5亿元，增长18.7%。

　　社会保障能力增强。就业规模进一步扩大，劳动关系保持和谐稳定。城镇新增就业4.4万人，比上年增长8.3%，城镇登记失业率为2.65%，全市领取失业金人数同比下降18%。企业职工最低工资标准上调至每月960元，增长24.7%。社会保障力度加大，基本社会保险参保人次达430.6万，增长13%，新型农村养老保险和城乡居民医疗保险实现全覆盖。城镇职工月均基本养老金达1728元，增加129元。加强最低生活保障工作，斗门农村低保标准提高40%。加快保障性住房建设，改造华侨农场职工危房2508户，提供廉租房、公租房252套，发放住房补贴308.2万元，解决了1508户困难家庭的住房问题。

　　文化建设全面加强。文化产业发展加快，实现增加值63.8亿元，比上年增长14.5%。文化设施建设加快推进，一批重大文化项目启动，一批基层文化站建成。新增省级文物保护单位7处。涌现一批文艺精品力作，获得省级以上大奖13项。文体活动蓬勃开展，我市体育健儿在广州亚运会上取得“8金2银”的好成绩。积极创建全国文明城市，加强全国城市公共文明指数测评迎检工作，群众性精神文明创建活动广泛开展，市民文明素质不断提高。

　　社会事业协调发展。制定《珠海市中长期教育改革和发展规划纲要（2010—2020年）》，不断提高教育现代化水平。新改扩建各类学校26所，加固中小学校舍41间。职业技术教育基地加快建设。各级财政安排补贴资金为全市14.6万名学生提供12年免费教育。高等教育健康发展，在校大学生增至11.5万人。积极创建国家健康城市，出台《珠海市健康城市规划纲要（2010—2015年）》，重大医疗基础设施、镇级薄弱卫生院改扩建和全市医疗就诊“一卡通”等项目建设加快推进。人口计生管理服务水平不断提高，连续22年圆满完成人口计生目标任务。人防、双拥、武装、国防教育工作取得新成效。外事、侨务、新闻出版、民族宗教、统计、海边防、海事、打私、打假、气象、科普、仲裁、档案、地方志、港澳流动渔民、老龄、红十字、关工委、老干、慈善和残疾人等工作取得新进步。

　　社会保持安全稳定。全市发展规模不断扩大，安全生产形势保持稳定。各类生产安全事故死亡人数比上年下降1.5%，工伤发生率控制在0.8%以下，亿元生产总值死亡率下降14.9%，严重犯罪案件发案同比下降29.3%，是珠三角社会治安、安全生产形势最好的城市之一。圆满完成上海世博会、广州亚运会、第八届中国航展等重大活动期间的安保工作。新建3个区级、23个镇街综治信访维稳中心，以及286个村（居）、24个规模以上企业综治信访维稳工作站，有效化解社会矛盾和不稳定因素。加大力度解决历史遗留债务问题，市财政安排资金偿还政府债务31.7亿元。加强应急管理，健全突发事件应急防控体系。食品药品、产品质量安全工作不断加强。香洲区荣获首批全国法治区创建活动先进单位。新增30个省级“六好”平安和谐社区，总数达143个。

　　（六）优化政务环境，服务型政府建设深入推进。

　　民主法制建设不断加强。全面推进依法行政，自觉接受市人大及其常委会和市政协的监督。全年办结市人大议案4件，办复代表建议158件、政协提案305件，办复率达100%。网络问政有序开展，科学民主决策机制不断完善。依法提请市人大常委会审议地方性法规4项，制定修改市政府规章7项。通过省“五五”普法验收，法治珠海建设深入推进。

　　行政服务水平持续提高。开展构建效能型机关、建设服务型政府活动，政府职能加快转变，政府执行力不断提高。加强行政服务中心建设，完善电子监察系统，全年审批和服务事项办结率达100%。基建项目审批流程不断优化，工程建设项目行政审批时间平均缩短50%。完善政府网站建设，政务公开工作深入推进。在省社科院的全省地方政府公共服务公众评价调查中，连续两年总体满意度名列第一。

　　廉洁从政工作扎实开展。加强制度建设，健全行政问责制，加大监察、审计工作力度，规范行政行为。全面落实党风廉政责任制，政府系统惩治和预防腐败体系建设深入推进。加强机关作风建设，民主评议政风行风活动深入开展，坚决纠正损害群众利益的不正之风。厉行勤俭节约，严格控制一般性行政经费支出，市直机关行政经费大幅降低。全市党政机关因公出国、出境人次比近三年平均数减少10%、支出下降30%，公务用车购置及运行费用下降10.8%，晚会展览庆典论坛活动减少13%。

　　各位代表，过去一年所取得的成绩，是省委、省政府正确领导的结果，是全市广大干部群众共同奋斗、辛勤劳动的结果，是海内外各界关心支持的结果。在此，我谨代表市人民政府，向珠海经济特区广大建设者，向给予政府工作大力支持的人大代表和政协委员，向各民主党派、人民团体、工商联和社会各界人士，向中央和各省（区、市）驻珠单位、驻珠军警部队，向所有关心和支持我市改革发展的港澳同胞、台湾同胞、海外侨胞和国际友人，表示崇高的敬意和衷心的感谢！

　　在肯定成绩的同时，我们也清醒地看到，在发展道路上还面临不少困难和问题：一是经济规模仍然偏小，进一步增强核心城市经济实力、有效发挥辐射带动作用的要求更加迫切。二是现代服务业发展还相对滞后，先进制造业整体竞争力还需进一步提高，内外源型经济发展还不平衡、不充分，产业结构性矛盾仍然突出，加快产业结构优化升级、构建现代产业体系的要求更加迫切。三是自主创新动力不足，企业的创新主体地位有待进一步强化，具有自主知识产权的自有品牌还不够多,加快建设创新型城市的要求更加迫切。四是工业化、城市化较快发展，能源、土地资源和环境的约束趋紧，促进全面协调可持续发展的要求更加迫切。五是城乡社会事业发展还不够平衡，强化全市上下“一盘棋”理念、统筹东西部地区协调发展的要求更加迫切。六是政府管理中还存在一些不足和薄弱环节，有的机关干部的发展意识、创新意识、责任意识和忧患意识有待强化，政府执行力和公信力有待提高，深入推进政府自身建设和政府职能转变的要求更加迫切。我们将进一步增强责任感、使命感和紧迫感，以更加扎实有效的措施解决发展进程中遇到的各种问题，切实做好政府各项工作，绝不辜负全市人民的厚望和重托！

　　二、“十一五”回顾和“十二五”展望

　　各位代表，2010年我市经济社会发展取得可喜成绩，为“十一五”划上了圆满的句号。过去五年，是我市发展进程中很不平凡的五年，是机遇重大、充满挑战、奋发有为的五年。五年来，全市人民团结奋斗，推动全市经济社会发展取得了新突破，开创了新局面。

　　——五年来，经济发展迈上新台阶。经济实力显著增强，2010年与2005年相比，全市生产总值、工业总产值分别增长78.2%和83%，年均增长12.3%和12.8%。来源于全市的财税总收入增长119.4%，年均增长17%。地方财政一般预算收入增长154.3%，年均增长20.5%。固定资产投资突破500亿元，增长129.5%，年均增长18.1%。产业规模不断壮大，形成“4+4+1”（高栏港经济区、高新技术产业园区、国家级航空产业园、富山工业园四大工业园区+南屏、三灶、新青、平沙四个特色产业园区+珠海保税区——珠澳跨境工业区一个特殊功能区）的工业园区布局，工业增加值增长91.4%，年均增长13.9%。新增规模以上工业企业409家，其中产值超亿元的299家。新增上市企业15家，总数达23家。经济增长的质量和效益明显提高，每平方公里土地产出生产总值和财政收入分别提高87.7%和152.4%。

　　——五年来，城乡建设呈现新面貌。港珠澳大桥、广珠铁路相继动工，广珠城轨建成通车，一批高速公路动工兴建，建设珠江口西岸交通枢纽城市的目标逐步实现。“十一五”时期交通投资累计完成180.4亿元，是“十五”时期的2.7倍。2010年与2005年相比，公路通车里程达1323.9公里，新增249.7公里，增长23.2%。高速公路通车里程达70.8公里，增长15.5倍。城市道路总长1410.1公里，增长45.1%。全市港口货物吞吐量达6056万吨，增加2500万吨，增长70.3%。全市口岸通关人数达9057.3万人次，增加1510.7万人次，增长20%。公共服务、市政环保等城市基础设施日趋完善，生态环境保持优良，园林绿地面积达5267万平方米，新增356.9万平方米，增长7.3%。城市水体和饮用水源水质达标率保持100%。

　　——五年来，民生事业取得新进步。2010年与2005年相比，六项民生支出占财政支出比重提高到39.6%，提高了14.7个百分点。人民生活水平持续提高，城镇居民人均可支配收入增长52.8%，年均增长8.9%。农村居民人均纯收入增长45.1%，年均增长7.7%。全市机动车总量达25万辆，增长63%；每百户居民拥有汽车31.5辆，增长1.6倍。城乡居民加快实现“住有所居”，人均住房面积达28.6平方米，提高0.94平方米。至2010年已累计改造华侨农场危房6133套，提供廉租房680套、公租房1500套，向1673户住房困难家庭发放租赁住房补贴939.6万元。就业形势稳定，城镇新增就业24.5万人次。教育协调发展，新增基础教育学位2.7万个，新改扩建学校65所，在全国率先实施12年免费教育，惠及学生101万人次。社会保障体系不断完善，全社会保险参保人次增加212万，增长97%，月人均养老金增长72%。率先建立全民医疗保障制度，将全市50多万外来务工人员及其子女纳入医疗保障覆盖范围，与本市居民享受同等医保待遇，基本医疗保险参保率达到97%。卫生事业加快发展，改扩建一批城乡医疗基础设施，建成128个农村卫生服务中心。

　　——五年来，改革开放实现新跨越。加快转变政府职能，创新政府管理，完成政府机构“大部制”改革，建立健全决策咨询、行政首长问责制、部门责任白皮书等80多项规章制度。建设“一站式”行政服务平台，推广应用网上审批、效能监察、公文处理等电子政务系统，机关作风建设深入推进，行政效能不断提高。完成平沙、红旗华侨农场体制改革，理顺区、镇管理关系，实施“镇场合一”、“区镇合一”的管理模式。研究处理了一大批历史遗留问题，切实保障群众合法权益。对外开放不断扩大，2010年与2005年相比，引进内资注册资本金增加72亿元，增长846.2%，年均增长56.7%。实际利用外资增加5.6亿美元，增长83.8%，年均增长12.9%。进出口贸易总额增加178.6亿美元，增长69.7%，年均增长11.2%。

　　通过五年的成功实践，我们深刻认识到：一是必须坚持科学发展。始终坚持抓好发展这个执政兴国的第一要务，积极探索建设生态文明的可持续发展新路。二是必须坚持改革创新。始终坚持发挥经济特区先行先试的创新创业创造精神，解放思想、改革创新，有效地突破发展瓶颈，破解发展难题，不断增强发展的全面性、协调性和可持续性。三是必须坚持民生共进。始终坚持正确处理改革发展稳定的关系，把维护最广大人民群众的根本利益作为一切工作的出发点和落脚点，执政为民，惠及民生，让人民生活持续得到改善。四是必须坚持真抓实干。始终保持清醒头脑，坚决落实中央和省的各项决策部署，坚持“干”字当先，结合实际创造性地开展工作，以真抓实干推动全市经济社会又好又快发展。五是必须坚持依法行政。始终严格按照法定权限和程序行使权力、履行职责，规范行政执法，完善行政监督，不断提高依法行政能力和政府执行力、公信力。所有这些，是过去五年我们形成的几点基本体会，也是我市再创新辉煌的宝贵财富。

　　未来五年，是关系我市建设核心城市、加快科学发展的关键五年，是我市率先转型升级、实现经济社会跨越发展的攻坚时期和重要战略机遇期。我们必须增强机遇意识、发展意识、大局意识、责任意识和忧患意识，以宽广的全球视野和敏锐的战略思维，深刻分析国内外形势，准确把握发展大势，牢牢抓住战略机遇，积极应对新的挑战，坚定不移地朝着宏伟目标阔步前进。

　　“十二五”时期政府工作的总体要求是：坚持走生态文明的发展道路，深刻把握科学发展主题和加快转变经济发展方式主线，以“率先转型升级、建设幸福珠海”为主旋律，深入实施珠三角《规划纲要》和《横琴总体发展规划》，大力推进交通、产业、城市三大格局建设，壮大经济实力，完善城市功能，优化生态环境，促进社会和谐，全面提升模式引领、实力带动、功能辐射能力，促进经济社会全面协调可持续发展，加快建设珠江口西岸核心城市和生态文明新特区、科学发展示范市。展望未来五年，我们的目标是：

　　（一）综合经济实力显著增强。珠三角《规划纲要》“四年大发展”目标任务全面完成，经济实力不断增强，经济结构更加优化，经济效益显著提高。全市生产总值比2010年翻一番，人均生产总值超过13万元。规模以上工业增加值超过1300亿元，形成3至4个年产值超百亿的产业集群。先进制造业增加值占规模以上工业增加值比重超过48%。战略性新兴产业增加值超过253亿元，占地区生产总值比重超过10%。现代服务业增加值占服务业增加值的比重超过 61%。年主营业务收入超百亿元企业4家，年销售收入超过100亿美元的本土跨国公司1家。财政一般预算收入超过240亿元，财税保障能力明显增强。

　　（二）珠江口西岸交通枢纽基本建成。机场高速、高栏港高速、金港高速建成通车，广珠铁路、广珠城轨自拱北经横琴至机场延长线建成营运。珠海大道完成改造。港珠澳大桥及连接线建设顺利推进。珠三角城际轨道江门至珠海机场段争取纳入珠三角规划并动工建设，完成市内轨道交通规划与评估。海港、空港功能更加完善，港口货物吞吐量接近2亿吨，机场旅客吞吐量超过500万人次。完成拱北口岸改造工程，口岸基础设施更加完善。现代化海陆空综合交通网络基本形成，区域性交通集疏运体系更加完善，城市交通更加畅通，市内除海岛以外的陆地区域实现15分钟内上高速公路。

　　（三）现代化区域中心城市初步形成。城市化和城市现代化进程加快，城市基础设施更加完善，城市规模进一步扩大。区域中心城市功能更加强化，产业和人口集聚能力提高，高端要素聚集发展功能和生产服务功能增强，城市综合服务水平和发展带动力、辐射力明显提升。做大做强中国国际航空航天博览会品牌，增强航展国际影响力。各城市组团功能布局优化，东部地区率先实现转型升级，科技创意、文化艺术、商贸旅游、商务行政等主题片区特色鲜明；横琴新区实现“五年成规模”目标，初步建成现代产业体系和生态型城区；西部中心城区开发建设取得重大突破，工业化与城市化发展更加协调。全市城市化水平达89%。

　　（四）自主创新能力明显提高。“创新驱动”战略深入实施，自主创新环境更加优化。大学园区资源优势充分发挥，产学研合作不断深化。国家重点实验室等创新平台不断完善，初步形成以企业为主体、市场为导向、产学研结合的开放型区域创新体系，自主创新能力显著提高，科技支撑引领作用显著增强。全社会研发（R＆D）投入占全市生产总值比重超过2.3%，成功申报2至5家国家级技术中心和工程中心，国家重点实验室等创新平台超过6家，年发明专利申请量每百万人口超过500件，高新技术产品产值占全部工业总产值的比重达45%。

　　（五）城乡区域发展更趋协调。充分利用经济特区范围扩至全市的契机，全面实施“绿色发展”、“东西互动”、“区域协同”战略。跳出传统约束、体制束缚和区划障碍，推动城市文化向农村发展、城市基础设施向农村延伸、城市社保向农村覆盖、城市公共服务向农村辐射、城市资源向农村倾斜，城乡区域一体化发展水平明显提高。全市户籍人口自然增长率控制在10.5‰左右。森林覆盖率达31%，城镇人均公园绿地面积达13平方米。城镇生活污水处理率达83%，工业废水排放达标率达100%。生活垃圾无害化处理率达100%。完成省下达的节能减排等约束性指标。

　　（六）人民生活更加幸福。“和谐共享”战略全面实施，建立经济特区发展成果惠及人民群众的长效机制。以改善民生为重点的社会建设全面加强，普惠型民生事业加快发展。人民群众生活更加殷实，富足感、安全感、公平感、归属感、自豪感不断增强。文化强市建设深入推进，文化事业繁荣发展，文化软实力显著提高。文化产业增加值占全市生产总值比重达8%。城镇登记失业率控制在3.5%以内，城镇基本养老保险参保率超过98%。继续实行十二年免费教育，城乡义务教育协调发展，高水平普及高中阶段教育，毛入学率接近100%，高等教育毛入学率达55%。社区卫生服务机构覆盖率100%。城镇居民人均可支配收入、农村居民人均纯收入明显提高。

　　（七）科学发展体制机制更加健全。经济特区改革“试验田”和先行先试作用充分发挥，经济体制、行政管理体制、社会管理体制改革不断深化，在重点领域和关键环节率先取得突破。政府职能加快转变，政府组织体系和运行机制、政府管理和服务方式不断优化，依法行政和公共服务的能力明显提高。农村经济体制、财政和投资体制、金融改革和创新、企业体制的深化改革成效明显，社会主义市场经济体系更加完善。社会管理综合改革取得重大突破，社会管理制度和社会管理方式不断完善，城市管理体制机制更为健全，形成具有珠海特色的社会管理模式。

　　各位代表，回顾过去五年，良好局面来之不易，应倍加珍惜；面向未来，新的征程任重道远，应倍加努力。当前，我们已经奠定了一个良好的发展基础；未来，我们必将续写珠海发展的崭新篇章！

　　三、2011年主要任务

　　2011年是建党90周年，也是我市实施“十二五”规划的起步之年和落实珠三角《规划纲要》“四年大发展”战略的关键一年，做好今年工作意义重大。综观国内外形势，今年仍是我市推动科学发展可以大有作为的一年，机遇与挑战并存，机遇大于挑战。一方面，国际金融危机影响仍在持续，世界经济复苏缓慢曲折，未来一个时期外需依然趋紧。美元持续贬值与人民币不断升值并存，原材料价格上升，国内通胀压力不断增大。我市用工紧张局面短时间内难以根本缓解，工业发展适度重型化的要求与节能减排约束趋紧之间的矛盾更加突出。所有这些都将使我们面临的形势更加复杂，挑战也更加严峻。另一方面，国家宏观经济环境整体趋于好转，内需不断扩大。我市一批重大交通基础设施加快建设，城市投资环境明显改善，为加快发展打下了坚实的“硬件”基础。珠三角《规划纲要》和《横琴总体发展规划》深入实施，横琴新区、高栏港区等重点区域加快开发建设，一大批工业项目开工建设和建成投产，为我市未来发展增添了巨大动力，等等。所有这些都是我们面临的重大利好和难得机遇，从根本上有利于我市率先转型升级，实现新一轮的跨越式发展。我们既要看到机遇，增强加快发展的信心和勇气，更要直面挑战，奋发进取，迎难而上，以对全市人民的深厚感情、以对特区事业的无限热情、以干事创业的奋发激情，投入到加快发展的各项工作中去，圆满完成今年的目标和任务。

　　统筹考虑各种因素，今年我市经济社会发展的主要预期目标是：实现地区生产总值增长11%左右；规模以上工业增加值增长13％；固定资产投资增长16％；外贸进出口总额增长10％；实际利用外商直接投资增长5%，引进内资注册资本金增长20％；财政一般预算收入增长13％；城镇居民人均可支配收入和农村居民人均纯收入分别增长10％和9.1％；城镇登记失业率控制在3.3％以内；户籍人口自然增长率控制在9.3‰以内；完成省下达的节能减排目标任务。

　　做好今年工作，要全面贯彻党的十七届五中全会、中央经济工作会议、省委十届八次全会以及市委六届九次全会精神，围绕发展“加速”目标，正确处理好总量增长与质量提升、经济发展与民生改善、改革发展与维护稳定三大关系，将率先转型升级、建设幸福珠海和全面落实珠三角《规划纲要》“四年大发展”任务贯穿于全市各项工作之中，着力推进交通、产业、城市、城市软实力和民生五大建设，努力促进全市经济社会又好又快发展，为“十二五”发展开好局、起好步。

　　（一）着力完善交通基础设施，加快构建珠江口西岸交通枢纽城市。

　　完善城市交通规划。抓紧推进《珠海市综合交通运输体系规划》编制工作。进一步完善港珠澳大桥连接线、连接东西城区的交通大动脉等重大交通规划，加强珠中江城际交通对接规划。抓紧编制市内轨道交通、快速公交和常规公交一体化的公共交通发展规划。结合西部中心城区开发建设，完善全市交通网络布局，进一步增强海港、空港的服务功能，加速构筑快捷、畅通、安全的城市交通体系。

　　加快交通项目建设。积极配合推进港珠澳大桥及连接线建设，加快高栏港高速、机场高速、金港高速金凤段（港湾大道至梅华立交）、井岸二桥等项目建设。争取广珠铁路珠海段今年底竣工、广珠城轨金鼎至拱北段今年内全线开通。金港路的翠屏段（梅华立交至造贝）、造贝连接线、南屏至横琴段（南屏至莲花口岸，含横琴二桥）和金海大桥开工建设，力争高栏港海底隧道、广珠城轨拱北经横琴到珠海机场段开工。加紧推进珠海大道主道、明珠路、港昌路改造，加快广珠城轨珠海各站周边路网建设。打通城市“断头路”，完善城区交通路网。建设道路交通配套设施，在上冲检查站路口等重点路口建设立交设施，加快解决停车难、人车争道等交通问题。启动全市快速公交系统项目，加快西部地区公交站场尤其是西部公交换乘中心建设。集中力量抓好高栏港区2个15万吨级矿石码头、1个10万吨级煤炭码头、4个10万吨级集装箱码头等项目建设。加快推动九洲港货柜运输功能有序转移，提升高栏港港口货物吞吐能力。

　　创新城市交通管理。坚持以人为本，加强交通组织、管制和疏导工作，提高路面见警率，缓解当前城区交通拥堵状况。开展道路交通秩序专项整治行动，加强各类机动车辆依法营运和安全行驶监管。加强城市道路及配套设施的维护管理，科学设置红绿灯、单行线和道路出入口。新建市政主要道路少设红绿灯，城市快速路不设红绿灯，千方百计提高道路通行能力。积极发展绿色公共交通，逐步推行电动公交车。建立完善城市道路养护维修工作巡检制度，强化市政工程设施管护的执法和监察工作。

　　（二）着力建设现代产业体系，加快转变经济发展方式。

　　大力推进重大项目建设。一是完善重大项目推进工作机制。建立储备、在谈、签约、在建和投产项目分类量化管理机制，以目标倒逼进度。健全层层抓落实的项目分工协调负责制，着力破解项目规划、审批、资金、用海、用地、供水、供电等方面的难题，以压力倒逼动力。坚持项目工作节点目标化管理，以日保旬、以旬保月、以月保年，以时限倒逼效率。加强督促检查和绩效考核评价，强化项目监督管理，打造优质工程、精品工程和廉政工程，以考核倒逼成效。二是加快推进在建项目建设。今年在建及年内开工项目80项，计划投资207亿元。加快协调推进港珠澳大桥等重大交通基础设施和中航通飞、中海油深水海洋工程装备制造、三一重工港口机械制造、玉柴柴油发动机等项目建设。想方设法推动已签约项目加快落地建设。三是统筹抓好中小型项目引进和服务。坚持长短结合、远近兼顾，在抓好大项目引进建设的同时，大力引进一批“两少、两有、两高”（占地少、用工少，有研发、有品牌，高技术、高效益）的中小型项目尤其是成长性强的科技型项目，提高产业配套水平和经济效益。出台鼓励和引导民间投资指导目录，加大民营企业扶持力度，拓宽民营资本投资渠道，加快发展民营经济。加强对中小企业的服务，切实解决“融资难”问题，支持中小企业发展。

　　大力提升产业发展水平。一是扩大增量，加大招商引资力度。围绕项目落户，以产业招商和项目招商为重点，做好投资推广、项目洽谈、跟踪服务等工作，以大招商促进大引进，实现大发展。积极参与我省面向大型央企、龙头民企和世界500强企业的三场重大招商活动，着力新引进一批投资大、产出高和成长性强的大项目、好项目。精心策划、积极有效地在国内外开展系列投资推广及小分队上门招商活动。全年实际利用外商直接投资额要超过12.8亿美元，引进内资注册资本金要超过96亿元。坚持引进项目与引进先进技术、先进管理、高端人才相结合，吸引各类国际高端人才汇聚，打造投资“洼地”。二是优化存量，改造提升传统产业。做强一般贸易，转型加工贸易，推动外商投资、加工贸易企业扩大内销。大力推进工业企业重点技术改造工程。出台政策措施提升专业镇发展水平，培育壮大建材家居、办公自动化及打印耗材等专业市场。在家用电器、服装等领域大力培育传统优势产业大企业集团。三是做大做强战略性新兴产业。抓紧完善相关产业发展规划和配套政策，重点培育50个战略性新兴产业项目，在高端新型电子信息、生物医药、新能源和新能源汽车3个产业领域取得突破。加快发展新材料、航空、海洋工程、节能环保4个产业。四是打造高端产业集群。加快建设海洋工程装备制造基地、航空产业园区和国际商务休闲旅游度假区。着重发展高栏港——富山沿海工业带和高新区两大工业生产片区，重点发展柴油发动机制造、石油化工、家电电气、生物医药、海洋工程装备、电子信息、清洁能源等产业集群。加强高新技术产业园区创新支撑服务体系建设，推进高新区“二次创业”，做优做精软件和集成电路设计、信息材料、电子通讯等高新技术产业集群。

　　大力发展现代服务业。着力推动服务外包、商务会展、金融、工业设计、现代物流、信息服务等生产性服务业做大做强，加快培育一批大型服务业企业。积极发展电子商务和物联网。壮大发展现代旅游、现代商贸等生活性服务业。整合旅游资源，推进旅游重大项目开发，大力开发海岛、温泉、观光农业等生态旅游项目，提升发展旅游业。举办2至3场大型文体旅游活动，拓展旅游综合消费。积极引进和建设大型商贸综合体，培育大型特色商业区，加快建设区域性商业服务业中心。组织2至3场珠货外销等大型展销活动，扩大珠海产品的影响力和美誉度。

　　大力推进自主创新。实施《珠海经济特区科技创新促进条例》，加快构建区域创新体系。加大自主创新投入，加快实施重大科技专项和重点技术攻关，重点培育50个自主创新项目。强化自主创新激励机制，完善支持企业创新的财税、金融和政府采购等政策体系。充分发挥企业主体作用，鼓励企业研发具有自主知识产权的技术产品。支持科技型中小企业上市融资，积极引进创业风险投资，促进科技与金融紧密结合。加强企业技术中心、工程中心、行业公共创新平台和重点实验室建设。继续深化产学研合作，推进省部产学研结合示范基地和产学研创新联盟建设，促进科技成果转化。深入开展质量强市活动，发挥质量奖导向作用，打造“珠海质量”品牌。加强知识产权工作，加大名牌名标产品培育力度。大力推进信息化建设，打造“智慧城市”。

　　（三）着力加快城市化进程，统筹城乡区域协调发展。

　　推进西部地区开发。高标准建设西部中心城区，加快起步区的土地资源整合和基础设施配套，把金湾、斗门区级中心和西部中心城区核心片区纳入起步区加快推进建设。坚持“同城一体、内外互动、协调发展”，进一步加大对西部地区的财政投入，加快东西部城市规划和基础设施、产业发展、义务教育、公共卫生、文化建设、城乡就业、社会保障、城市管理等方面的一体化进程。加快完善工业园区生活配套设施，大力支持高栏港区、航空产业园和富山工业园等重点园区发展。

　　提升发展东部地区。东部地区要大力发展现代服务业，加快发展总部经济，提升服务业聚集区发展水平。引进建设1至2个国际高端品牌酒店。大力推进十字门中央商务区、拱北口岸地区商业中心改造等项目建设。落实“退二补公进三”（退出第二产业，补充公共绿地和公共服务设施，发展现代服务业）措施，推进香洲主城区旧厂房、城中旧村改造，提升东部城区建设水平。推动高新区加快完善生产生活配套设施，实现提速发展、争先进位。加大海洋综合开发力度，积极推进万山区创建国家级海洋综合开发试验区。加快保税区和跨境工业区转型升级。

　　促进“三农”发展。加大财政投资和信贷资金对农村农业的投入，今年全市财政安排“三农”资金30.6亿元，进一步提高农业产业化、集约化水平。加快推进城乡水利防灾减灾工程建设，完成白蕉联围和中珠联围加固达标工程，加快推进斗门四小联围海堤达标建设，开展白龙河水闸前期研究，完善农田水利基础设施。开展村庄整治，绿化、美化城乡环境。选取斗门区1个镇和横琴新区、金湾区、斗门区、高新区、高栏港区各1个村，作为统筹城乡一体化发展示范镇、村，进行重点规划建设，确保一年初见成效。建设1至2个名镇、名村，示范带动宜居城乡建设。做好村（居）两委换届工作，扶持发展村集体经济，加快建立农民增收的长效机制，实现农村居民人均纯收入超过1.1万元。支持斗门北部生态农业园创建国家级生态农业园，抓好台湾农民创业园建设，发展现代生态农业。落实“菜篮子”行政首长负责制，加强农产品检测体系建设，强化农产品质量安全工作。调整优化渔港功能布局，发展现代渔业。

　　深化区域合作。深入推进珠港澳更紧密合作，加大横琴新区开发建设力度，提升开发热度。落实横琴新区体制机制创新要求，抓紧优惠政策报批工作，加紧完善基础设施配套，加快产业发展，增强生产要素集聚功能和自我发展能力，实现“两年见成效”建设目标。加快竹银水源工程建设，确保今年4月主体工程完工。加快推进我市供水保障双回路工程建设。深入推进珠中江合作，加快落实三地已签订的36项合作协议，进一步扩大合作成果。积极推进“双转移”工作和扶贫开发“双到”工作。扎实做好支援四川凉山地区等对口帮扶工作。

　　（四）着力提升城市软实力，增强核心城市集聚和辐射能力。

　　加快建设文化强市。今年新增文化发展经费2800万元，促进文化事业发展。健全公共文化服务体系，加快推进“一院三馆”（市歌剧院、博物馆、城市规划馆、文化馆）、横琴粤港澳文化产业园和珠海南方影视传媒等项目建设。加快培育文化创意产业，支持有技术、有品牌、有市场、有潜力的动漫企业做大做强。深入开展全国文明城市创建活动，切实抓好各项创建工作落实，提高市民参与度。加强历史文化资源的综合保护与开发利用，积极创建国家历史文化名城。支持文艺精品创作，最大限度满足人民群众文化需求。

　　加大人才工作力度。加强招才引智工作，加大人才资源开发力度。积极引进海内外创新科研团队和领军人才，支持创新科研团队的建设。发挥科技重奖的导向作用，激励科技人才创新创业。充分发挥各类教育培训机构作用，培养经济社会发展紧缺人才。完善人才政策，优化人才工作和生活条件，切实解决人才住房、医疗、社保和子女入学等方面的实际问题，营造事业留人、待遇留人、环境留人和人尽其才的良好氛围。

　　推进改革攻坚。坚持在重点领域和关键环节先行先试，加快形成有利于科学发展的体制机制。巩固“大部制”改革成果，完善政府运行机制。稳步推进事业单位分类改革，深入推进富区强镇、医药体制等重点领域改革。促进市属投融资平台转型升级，发展混合所有制经济。推进财政体制改革，完善预算编制和执行监管制度，优化财政支出结构，建立适应基本公共服务均等化和主体功能区建设要求的公共财政体系。推进金融改革创新，利用国家多层次资本市场，加快自身发展。深入推进社会管理体制改革先行先试。加强城市信用体系建设。

　　优化生态环境。深入实施生态建设“四个百分百”行动方案，加快建设国家生态文明试点城市，积极创建低碳城市。按照国家和省的要求完成国家环保模范城市复查迎检项目建设。实施“森林城市、生态家园、幸福珠海”建设工程，推进重点生态工程、绿道网和城市公园建设。落实节能减排目标责任制，突出抓好工业、建筑、交通、公共机构等重点领域节能工作。继续完善排污自动监控系统，大力推动结构性减排。深入治理土壤、水、大气和固体废弃物污染。加快推进海岛垃圾和污水处理工作，加强海洋生态环境保护。大力推进高栏港热电联供项目和循环经济示范园区建设。发挥土地利用规划的管控作用，加大国土资源执法力度，加强闲置土地清理和土地储备，严格保护耕地，强化节约集约用地。落实自然资源有偿使用和生态补偿机制。开展“城市管理年”活动，以香洲区为主战场，市、区、镇（街）、社区四级联动，重点整治城市脏乱差、交通拥堵、烟尘噪音污染等突出问题，营造“净、畅、宁、美”的城市环境。

　　加强民主法制建设。做好依法治市工作，深入推进依法行政，营造良好的法治环境。自觉接受监督，进一步完善科学决策机制。加强行政服务中心建设，创新和拓展政府服务的内容和方式，突显政府服务的新特色、新优势。全面推进政务公开，向社会公布政府部门责任白皮书和主要职能、审批事项及审批要求，提高政府决策透明度。大力推进反腐倡廉制度建设，落实行政问责制。加强审计监督和行政监察，强化经济责任审计和绩效审计。加大公务员培训力度，提高公务员队伍的整体素质。办好人大议案、代表建议和政协提案。

　　（五）着力改善民生，促进社会和谐。

　　改善民生福利。加大民生投入，全面推进惠民生办实事30项工程建设，保质量、保安全、保廉洁。采取强有力措施稳定消费价格总体水平，保障群众基本生活。实施全民职业能力提升计划和创业带动就业工程，加强外来务工人员和大学毕业生就业服务，稳定和扩大就业。加强企业人文关怀，完善劳动关系调处机制，促进劳动关系和谐稳定。进一步健全社会保障体系，提高社会保障能力。加强房地产市场调控，做好住房保障工作，进一步改善中低收入群体的住房条件。

　　发展社会事业。大力推进义务教育规范化学校建设，全市规范化公办中小学达到100%。深入实施校安工程，确保校园安全。突出抓好学前教育，大力发展职业技术教育，加快推进职教基地建设，办好珠海城职院，提高高等教育办学水平。深入实施《珠海市健康城市规划纲要（2010—2015年）》，加强重大疾病防控、医疗救治和卫生监督体系建设，完善职业病防控体系，通过残疾人“人人享有康复服务”国家评审验收，加快建设健康城市。进一步加强人口计生工作，稳定低生育水平，深入推进人口均衡型社会建设。完善公共体育设施，深入开展全民健身运动。促进其他社会事业全面发展。

　　维护社会和谐稳定。坚决落实维稳工作责任制，加强社会管理工作，强化社会治安综合治理，大力整治突出治安问题和突出治安地区，积极预防和妥善处置群体性事件。着力加强安全生产工作，严格落实“一岗双责”责任制（“一岗”是指党政领导干部职务对应的岗位；“双责”是指领导干部既要履行岗位业务工作职责，又要履行安全生产工作职责），切实抓好重点行业、重点领域的安全监管和隐患排查整治。深入开展产品质量和食品药品安全专项治理。完善应急管理体系，提高预防和处置各类突发事件能力。

　　各位代表，“十二五”规划是我市未来五年经济社会发展的行动指南。今年是实施规划的第一年，我们要高度重视，精心组织，狠抓落实。实行目标责任管理，逐级分解下达规划目标任务和重点工作。制定实施规划的配套政策、工作方案和年度计划。建立跟踪督查和考核评估机制，实施并强化行政领导负责制和责任追究制，确保规划的各项目标任务落实到位，全面完成。

　　各位代表，在这里，我代表市人民政府郑重承诺，今年还要办好人民群众关心关注的几件民生实事：一是新建市文园中学新校区、市第十一中学和斗门特殊教育学校，年内基本建成11个镇中心幼儿园。二是在全市范围内实施外来务工人员随迁子女凭积分免费入读我市义务教育公办学校办法，进一步扩大十二年免费教育覆盖面。三是城镇新增就业4万人，农村劳动力转移就业4000人，帮助1800名困难家庭人员实现就业；投入2000万元建立城镇居民养老保险制度，推进城乡居民社会保障一体化。四是加快普惠型社会养老服务体系建设，基本完成西部地区10所镇级福利机构改扩建工作；为200名符合条件的老年人提供居家养老服务；安排1584万元为全市80周岁以上的老年人发放高龄老人津贴、为“三无”及五保对象发放节日慰问金、生活补助和物价补贴。五是实施医疗就诊“一卡通”，完成5个薄弱卫生院改造，从今年起市财政安排资金连续三年每年培训100名社区全科医生，优化基层医疗卫生服务体系，提高公共医疗卫生服务水平。六是建设保障性住房4801套，其中廉租房260套、改造危房2495套、公租房2046套，提高住房保障服务能力。七是新建一批人行过街设施；建设一批停车场，新增1384个停车位，缓解城区停车难问题。八是建成平沙、新青、白蕉、富山4座污水处理厂；开展垃圾分类处理设施建设，加快西部固废无害化处理中心应急填埋工程和旧填埋场封场工作。九是完成斗门、金湾、高新区69个农村、社区的水改工程；投入6200多万元建设农村公路，实现62个200人以上自然村通水泥路，总里程约44公里；推行公交车同城同价，促进东西部地区公交事业协调发展。十是建设4至5个水产养殖、优质蔬菜、畜牧养殖等大型农产品生产基地，丰富蔬菜、肉类等产品供应，维护农产品价格稳定。

　　各位代表，宏伟蓝图已经绘就，发展前景催人奋进。迈步在新的征程上，我们深感肩负的使命艰巨而光荣。让我们紧密团结在以胡锦涛同志为总书记的党中央周围，高举中国特色社会主义伟大旗帜，以邓小平理论和“三个代表”重要思想为指导，深入贯彻落实科学发展观，在市委的正确领导下，团结一心，开拓进取，扎实工作，为开创珠海经济特区更加美好的明天而努力奋斗！
